# Hybrid Attention Cascade Network for Facial Expression Recognition

**DOI:** 10.3390/s21062003

**Published:** 2021-03-12

**Authors:** Xiaoliang Zhu, Shihao Ye, Liang Zhao, Zhicheng Dai

**Affiliations:** 1 National Engineering Laboratory for Educational Big Data, Central China Normal University, Wuhan 430079, China; zhuxl@ccnu.edu.cn; 2 National Engineering Research Center for E-Learning, Central China Normal University, Wuhan 430079, China; yeshihao@mails.ccnu.edu.cn (S.Y.); dzc@ccnu.edu.cn (Z.D.)

**Keywords:** facial expression recognition, attention cascade network, ResNet, GRU, AFEW

## Abstract

As a sub-challenge of EmotiW (the Emotion Recognition in the Wild challenge), how to improve performance on the AFEW (Acted Facial Expressions in the wild) dataset is a popular benchmark for emotion recognition tasks with various constraints, including uneven illumination, head deflection, and facial posture. In this paper, we propose a convenient facial expression recognition cascade network comprising spatial feature extraction, hybrid attention, and temporal feature extraction. First, in a video sequence, faces in each frame are detected, and the corresponding face ROI (range of interest) is extracted to obtain the face images. Then, the face images in each frame are aligned based on the position information of the facial feature points in the images. Second, the aligned face images are input to the residual neural network to extract the spatial features of facial expressions corresponding to the face images. The spatial features are input to the hybrid attention module to obtain the fusion features of facial expressions. Finally, the fusion features are input in the gate control loop unit to extract the temporal features of facial expressions. The temporal features are input to the fully connected layer to classify and recognize facial expressions. Experiments using the CK+ (the extended Cohn Kanade), Oulu-CASIA (Institute of Automation, Chinese Academy of Sciences) and AFEW datasets obtained recognition accuracy rates of 98.46%, 87.31%, and 53.44%, respectively. This demonstrated that the proposed method achieves not only competitive performance comparable to state-of-the-art methods but also greater than 2% performance improvement on the AFEW dataset, proving the significant outperformance of facial expression recognition in the natural environment.

## 1. Introduction

Emotion recognition systems are helpful in human–machine interactions [1]. Physiological signals, facial features, speech features, etc. are usually used to extract emotional features. Among them, the human face is considered an intuitive and non-intrusive biometric trait for automatic human authentication. It is also common for people to use facial expressions to communicate and express emotions. Therefore, automatic facial expression recognition (FER) has significant application potential to improve human–computer interaction. Ekman et al. divided facial expressions into six basic categories (happiness, sadness, surprise, anger, fear, and disgust) [2]. However, facial expression is a dynamic and continuous process that changes from the beginning, to the peak, and then to the end [3]. Traditional expression recognition methods, such as principal component analysis (PCA) [4,5], Gabor wavelet [6], and local binary pattern [7], use static images for recognition. These methods only consider the expression at the peak and ignore the influence of dynamic changes [8]; therefore, researchers have gradually shifted their focus from static image recognition to dynamic video sequence recognition [9]. Currently, FER methods using video sequences have achieved good accuracy in experimental environments; however, most of the facial expressions appear under natural conditions rather than through emotion induction experiments [10]. In natural environments, facial expressions will be affected by various factors, such as head deflection, facial posture, uneven illumination, and blur [11]. Therefore, automatic FER in natural environments remains challenging; thus, it is also one of the key issues in designing natural human–computer interaction (HCI) [12,13]. Recent deep neural networks-based methods have achieved state-of-the-art performance on various FER tasks [14]. The traditional two-dimensional (2D) convolutional neural networks (CNN) has a relatively low computational cost; however, it cannot capture the time relationship. The three-dimensional (3D) CNN-based methods can achieve good performance. However, the calculation cost is significant; thus, the deployment cost is high. Therefore, a universal and effective time conversion module, which has both high efficiency and high performance, should be developed to meet new challenges [15]. The contextual relationship among consecutive images plays an important role in improving recognition performance. Therefore, space–time correlation methods have been investigated [15,16]. Due to their promising ability to learn discriminative features, visual attention mechanisms are increasingly used to address pattern recognition problems. Such problems include automatically locating critical facial regions to eliminate the influence of irrelevant facial parts and fusing diversified attention to learn discriminative features [17,18].

Based on the above considerations, in this paper, we propose a cascaded neural network method for FER from dynamic video sequences in natural environments. The primary contributions of this study work are as follows.

The spatial and temporal features of facial expressions are extracted using a Residual Neural Network (ResNet) and a Gated Recurrent Unit (GRU) cycle unit. A hybrid attention mechanism is added to the cascaded network to obtain the association between expression frames and extract the attention feature weight of the face to improve the recognition ability of the proposed method in the natural environment. Finally, we collect the facial expression public datasets from two different environments (the natural environment and the experimental environment) to analyze the proposed method and then verify the robustness of it using different evaluation indexes.

The remainder of this paper is organized as follows. Section 2 introduces previous studies related to FER. Section 3 provides an overview of the proposed method as well as describes the modules and overall framework of the model. The experimental process, including the datasets used, pretreatment, parameter settings, and an analysis of the experimental results, are discussed in Section 4. The Conclusion is provided in Section 5.

## 2. Related Work

### 2.1. Facial Expression Recognition Methods

Typically, facial features are extracted by the handcrafted feature extraction, such as principal component analysis (PCA) [4,5], wavelet transform [6], local binary patterns (LBP) [7], etc. On one hand, the handcrafted feature extraction of such methods is primarily based on a lot of experimental experience; thus, it is inefficient. The most traditional methods are designed for specific recognition tasks; therefore, there are obvious differences in FER conditions, e.g., illumination, facial pose, blur, and race, in different environments [19].

With the development of deep learning technology, many methods have been applied to solve the FER problem, which are primarily divided into static image-based and dynamic video sequence-based methods [8]. In the static image-based method, Mollahoseini et al. applied a convolutional neural network to FER [20]. Additionally, Qin et al. achieved good recognition effect on multiple datasets using ResNet [21], and Yao et al. designed the HoloNet network and constructed the middle layer using a residual structure and CReLU (Concatenated Rectified Linear Units) to improve the efficiency of the deep network [22]. Additionally to a single RGB image as the network input, researchers have also combined geometric features, appearance features, facial motion units, and other factors of the face to form multimodal features, and then inputted these features into the network to enhance network performance. For instance, Cui et al. proposed systematically capturing dependencies between facial expression and action units (AUs) and incorporate them into a deep learning framework [23]. Liu et al. proposed combining acoustic features and facial features in both non-temporal and temporal mode [24]. Zeng et al. defined a high-dimensional feature composed by the combination of the facial geometric and appearance features [25]. Ding et al. presented FaceNet2ExpNet to train an expression recognition network based on static images [26].

The static image-based method typically only consider the expression recognition of a single image and ignores the temporal relationship of images in the sequence [8]. In fact, dynamic changes in facial expressions are important information for FER. In the dynamic video sequence-based method, some space–time correlation methods are proposed, such as latent ordinal model (LOMo) [27], deeper cascaded peak-piloted network (DCPN) [28], peak-piloted deep network (PPDN) [29], etc. Emotiw2015, which is an expression recognition challenge in the natural environment, has achieved better classification results than a network using only a CNN by introducing a recurrent neural network (RNN) [30]. Accordingly, some studies have combined CNNs and RNNs to solve the FER problem. By cascading CNN and RNN networks, facial expressions in video can be classified. Here, features are extracted by the CNN, and the extracted features are input into RNN training to estimate the expression classification of the entire video [31,32]. Cai et al. used a cascaded network of a CNN and bidirectional long short-term memory (LSTM), and they completed the FER task in a natural environment through dual channel fusion (audio and visual) [33]. Additionally to the RNN, 3D convolutional networks are often used in FER. Compared to traditional 2D convolution, 3D convolutional neural networks include a kernel on the time axis to form a 3D convolution kernel to capture the temporal and spatial characteristics of video sequences. In the FER task, a 3DCNN and its derived network structure have demonstrated excellent recognition effect [34]. Pini et al. applied C3D (convolutional 3D) as a feature extractor to obtain multichannel static and dynamic visual features and audio features, and they fused the network to extract spatial–temporal features [35]. Hasani et al. obtained the Hadamard product between a facial feature point vector and a feature vector in an inflated 3D convolution network (I3D) and cascaded an RNN to realize end-to-end network training [19].

### 2.2. Attention Mechanism

The attention mechanism has been widely used in machine translation, image classification, and other fields [36]. The important characteristic of the attention mechanism in computer vision is that it does not process the entire scene in an image. Instead, it calculates the attention weight through a feature map and selects the focus area of the image via weight distribution to capture more critical visual features [37].

FER tasks are increasingly applied to attention mechanisms. For example, Meng et al. proposed Frame Attention Networks for video-based facial expression recognition, which contains a self-attention module and a relation-attention module [18]. Zhao et al. added spatial attention and channel attention into a convolutional neural network to construct a regression model to analyze fine-grained visual emotion [38]. Additionally, Gera et al. proposed a spatial channel attention network (SCAN), which can obtain global and local attention for each channel and the spatial position of the expression sequence to recognize a facial expression with posture change and occlusion, and they verified that recognition accuracy was improved in multiple natural environment datasets [39]. Lee et al. proposed a multimodal recurrent attention network (MRAN) combined with image color, depth, and a thermal map for facial expressions under different skin colors and environments to learn the attention weight through three channels, and they supplemented feature information missing based on an attention-enhanced feature vector [14]. Additionally, Sun et al. proposed a dynamic sequence FER system that integrates shallow and deep features with the attention mechanism [40]. However, the accuracy that was obtained on an AFEW (Acted Facial Expressions in the wild) dataset was unsatisfactory. Gan et al. presented a relatively complicated mechanism that adopted a multiple attention network that simulates human coarse-to-fine visual attention to improve expression recognition performance; however, the performance of this method on CK+ (the extended Cohn Kanade) was not ideal, and performance was not validated on the AFEW datasets [17].

## 3. Proposed Methodology

### 3.1. Method Overview

**Figure 1:**
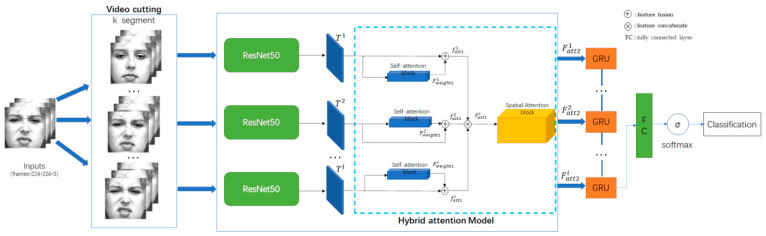
Structure of proposed hybrid attention network model. We note that *k* represents segments of frames; $T^{i}$ represents the spatial feature of the *i*th frame extracted by ResNet50; $F_{weight1}^{i}$ represents the self-attention feature vector; $f_{att1}^{i}$ represents the outputs by feature fusion operation; $\;F_{att1}^{i}$ represents the combined output by feature concatenate operation; $F_{att2}^{i}$ represents the feature vector of the hybrid attention module; FC represents the fully connected layer; +represents the feature fusion operation; ×represents the feature concatenate operation.

We proposed a hybrid attention mechanism to recognize facial expressions in video sequences. The structure of the model is shown in Figure 1. We divide the processed video sequence into *k* parts, randomly extract a frame from each part, and use the extracted *k* frame sequence *F* as the input to the network to extract features.

(1) $$F=\left\{f^{1},f^{2},\ldots ,f^{k}\right\},\;\left(k\in N\right)$$

here, *f^k^* is a randomly selected frame from each part, and *N* is the total number of frames in the processed video sequence. We note that k is decided based on *N*. For example, in our study, k was set to be 5, because the average value of *N* in the videos we used is 5, i.e., *k* = mean(*N*). (1) In the case when N ≥ 5, (i.e., the total number of frames in a video is equal or greater than 5), the video sequence is divided into 5 parts; in each part, one frame is randomly selected and taken as one of the 5 extracted frames for further processing; (2) in the case when N < 5, all the available frames are taken as the extracted frame.

The network is divided into three modules, i.e., the spatial feature extraction module, the temporal feature extraction module, and the hybrid attention module. The spatial feature extraction module selects the residual network as the spatial feature extractor and inputs the extracted spatial features to the hybrid attention module. In the hybrid attention module, attention weights are learned via self-attention, spatial attention, and specific feature fusion methods, and then they are weighted into the spatial feature vector to form hybrid attention features. The temporal feature extraction module adopts the weighted hybrid attention feature vector to extract the temporal features using the gating cycle unit GRU. The temporal feature extraction module is connected by a fully connected layer and is input to a SoftMax layer to output the classification results.

### 3.2. Hybrid Attention Module

The hybrid attention module calculates the feature weight of the expression using the attention mechanism. It assigns a higher weight to an attention area with expression change. In contrast, less weight is assigned to a region that is unrelated to expression change to allow the network to learn the features of the attention area, eliminate irrelevant features in the video, and improve recognition accuracy in the natural environment.

**Figure 2:**
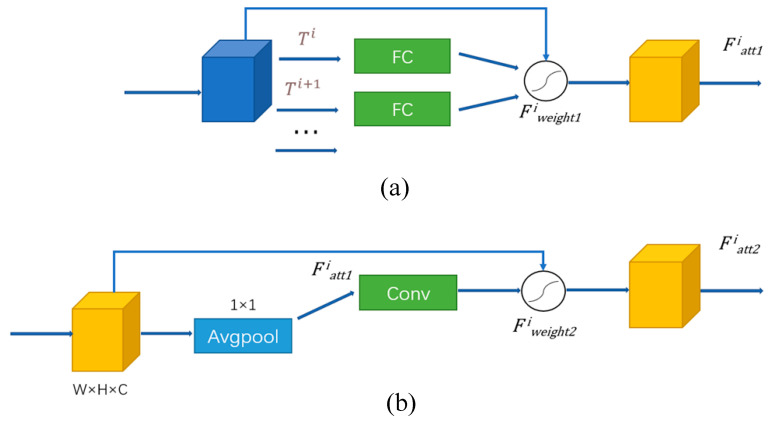
Hybrid attention module structure: (**a**) self-attention module; (**b**) spatial attention module.

The hybrid attention module comprises the self-attention and spatial attention modules. The module structure is shown in Figure 2. Here, the spatial features are first introduced into the self-attention module, the attention distribution of single frame features is calculated, and the feature vector $F_{att1}^{i}$ of the self-attention module is obtained.

The self-attention module is realized by a fully connected layer and an activation function, sigmoid. As shown in Figure 2a and Equation (2), first, $T^{i}$, which is the spatial feature of the *i*th frame extracted by ResNet50, is input into a fully connected layer in the self-attention mechanism to obtain the self-attention weight θ. Second, $T^{i}*\theta$ is put into a sigmoid activation function. Finally, the output spatial features are aggregated to obtain the self-attention feature vector $F_{weight1}^{i}$.

(2) $$F_{weight1}^{i}=\sigma \left(T^{i}*\theta \right)$$

here, σ is the sigmoid activation function. The spatial characteristics $T^{i}$ and $F_{weight1\;}^{i}$ perform feature fusion to obtain feature vector $f_{att1}^{i}$ output from the self-attention module.

(3) $$f_{att1}^{i}=\frac{\sum_{i=1}^{k}F_{weight1}^{i}T^{i}}{\sum_{i=1}^{k}F_{weight1}^{i}}$$

Subsequently, we combined the outputs of self-attention modules as follows.

(4) $$F_{att1}^{i}=\left[f_{att1}^{1},f_{att1}^{2},\ldots f_{att1}^{k}\right]$$

where $f_{att1}^{1},f_{att1}^{2},\ldots f_{att1}^{k}$ represent the 1st, 2nd, …, *k*th outputs of self-attention modules, and $F_{att1}^{i}$ represents the combined output. In our study, this process was conducted by torch.cat (dim = 1), where torch.cat is a function in Pytorch concatenating the given sequence of tensors along the given dimension.

The attention weight extracted by a single frame ignores the information association between frames; therefore, we include the spatial attention module after upper processing and make feature vector $F_{att1}^{i}$ of the self-attention module input to the spatial attention module to extract the feature weight of the spatial information of video sequence. The spatial attention module is shown in Figure 2b. This module passes through a 1 × 1 average pooling layer, extracts the spatial attention weight ω via a 2D convolution layer (with kernel size 3 × 3 and padding size 1), and finally calculates spatial attention feature vector $F_{weight2}^{i}$ using the sigmoid activation function. We note that the purpose of putting a 1 × 1 average pooling layer before the convolution layer is to extract the global image features.

(5) $$F_{weight2}^{i}=\sigma (F_{att1}^{i}*\omega )$$

Finally, we obtain eigenvector $F_{weight2}^{i}$ from the spatial attention module, and input feature $F_{att1}^{i}$ performs further feature fusion to obtain the final output feature vector $F_{att2}^{i}$ of the hybrid attention module.

(6) $$F_{att2}^{i}=\frac{\sum_{i=1}^{k}F_{weight2}^{i}F_{att1}^{i}}{\sum_{i=1}^{k}F_{weight2}^{i}}$$

Feature vector $F_{att2}^{i}$ obtained by the hybrid attention module contains the association information between frames. The feature weight is weighted to the spatial feature vector by two feature fusion processes to extract the target features. By learning the target features, the network can effectively eliminate the influence of the natural environment, e.g., lighting and face deflection. 

### 3.3. Network Model Structure

The proposed hybrid attention network model is a cascaded network comprising the spatial feature extraction, hybrid attention, and temporal feature extraction modules. The spatial feature extraction module uses the depth residual network ResNet50 structure. In deep learning, a network with deep network layers typically faces gradient disappearance or gradient explosion problems. A deep residual network employs a unique residual block in its structure to perform identity mapping on the features, and the input features of the current layer are transferred to the next layer structure. Additionally, the shortcut connection does not produce additional parameters and will not increase computational complexity. Thus, the deep residual network can effectively prevent performance degradation by deepening the deep convolutional neural network layers. Simultaneously, the batch normalization [41] and dropout [42] layers in the network effectively prevent the model from overfitting and address gradient disappearing. Table 1 details the network structure of each layer of the ResNet50 model.

Therefore, it is necessary to further extract temporal features from hybrid attention features. Here, the temporal feature extraction module selects the gating cycle unit GRU as the network structure. Compared to other cyclic neural network structures, the GRU unit model is simpler, especially in a deeper network model. The GRU can forget and select memory simultaneously via a single gate control. The number of parameters is greatly reduced and efficiency is higher. Through experimental comparison, we found that the recognition rate of the GRU network with 128 neural units in the single-layer hidden layer was best; thus, we employ a GRU with 128 neural units in the single-layer hidden layer as the temporal feature extraction network.

## 4. Experiment and Results

### 4.1. Datasets

In this study, we used the AFEW [43], CK+ [44,45], and Oulu-CASIA (Institute of Automation, Chinese Academy of Sciences) [46] datasets in our experiments.

**Figure 3:**
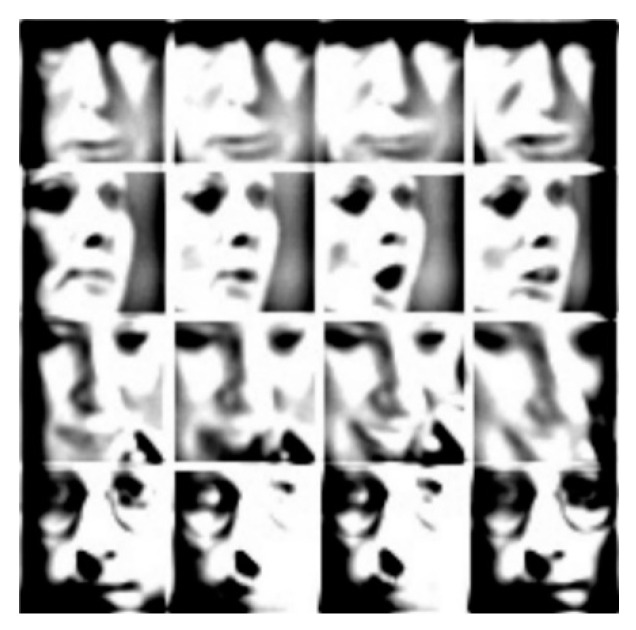
Complexity diversity of AFEW (Acted Facial Expressions in the wild) datasets.

The AFEW dataset collecting spontaneous emotion clips from movies or TV plays is the EmotiW emotion recognition challenge dataset. It contains 773 training set samples, 383 verification set samples, and 653 test set samples. Each sample takes one of seven labels, i.e., neutral, anger, disgust, fear, happiness, sadness, or surprise. The complexity and diversity of the AFEW dataset is shown in Figure 3 (To protect the privacy, the resolution of this figure is reduced). In this dataset, the video content was taken from a natural environment; thus, the face is affected by various factors, e.g., head posture, deflection, illumination, and video resolution; thus, it is challenging to use this dataset for expression recognition tasks. 

The CK+ dataset is a facial expression dataset collected by a team at the University of Pittsburgh. This dataset contains 593 facial expressions of image sequences, and each image sequence contains 10 to 60 frames, where the expression changes gradually increase from neutral to peak. Among the 593 image sequences, 327 include expression tags. In this study, 327 image sequences with expression tags were selected as datasets. The tags were divided into seven basic expressions, i.e., anger, contempt, disgust, fear, happiness, sadness, and surprise.

The Oulu-CASIA dataset is a facial expression dataset jointly released by the University of Oulu and the Chinese Academy of Sciences. This dataset contains six expressions, i.e., surprise, happiness, sadness, anger, fear, and disgust, from 80 people (50 Finnish and 30 Chinese; aged 23 to 58). This dataset contains image sequences under three illumination conditions, i.e., normal illumination, weak illumination, and no light scenes.

### 4.2. Data Preprocessing

In different environments, a facial expression can be affected by lighting, head posture, environment, and other factors, which makes the performance of different images in the network differ significantly. Preprocessing a dataset can effectively eliminate redundant information and reduce data noise, thereby improving the effectiveness of facial expression feature extraction. Therefore, prior to network training, data preprocessing is essential.

In this study, the AdaBoost algorithm based on Haar features [47] was used to calculate the gray-level change of an image initially. Through the pixel region difference, face detection was performed on the dataset, and the face image in the image sequence was extracted. In a natural environment, detected faces may have different angles according to the head posture; thus, to ensure that the input has less interference in the network, face alignment is performed in the sequence-based on the detected face images [48]. Here, the depth network feature detector, *Ensemble of Regression Trees*, provided by the dlib tool library was used to detect 68 facial feature points, including eye, eyebrow, nose, mouth, and facial contour. According to the point information of the 68 extracted facial feature points, the information of the middle face point was calculated. Based on the position information of the first frame of the video sequence, an affine transformation matrix was used to adjust the angle of the subsequent image sequence to achieve face alignment.

Finally, the face image was adjusted to 224 × 224 pixels and normalized. Here, the CK+, AFEW, and Oulu-CASIA datasets were tested via five-fold cross-validation. Four groups of data were selected as a training set, one group of data was used as a validation set, and the average classification accuracy rate was obtained after five training processes as the final verification accuracy rate.

### 4.3. Network Training and Experimental Parameter Settings

The network model was based on the Pytorch deep learning framework. The experimental environment used an Ubuntu16.04 system, and the hardware included an Intel i7-6800k CPU and an NVIDIA GTX1080ti GPU. In the network training stage, the dataset sample label was transformed into one-hot coding form. Additionally, the SGD optimizer with random gradient descent was employed, and L2 regularization was implemented to prevent model overfitting. The sigmoid function was used as the activation function, the weight attenuation was set to 0.0001, momentum was set to 0.9, the batch size was set to 16, and the cross-entropy loss function was employed.

(7) $$loss=-\frac{1}{N}\underset{i}{\sum }\overset{M}{\underset{c=1}{\sum }}y_{ic}\log\left(p_{ic}\right)$$

where *i* represents the *i*th sample, *c* represents the predicted label, and $p_{ic}$ represents the probability that the predicted label of the *i*th sample is C.

Different learning rates and epochs were set for different datasets. Here, the learning rate was set to 0.001 for the CK+ dataset, and 100 epochs were iterated. The learning rate was set to 0.0001 for the AFEW dataset, and 200 epochs were iterated. The learning rate was set to 0.001 for the Oulu-CASIA dataset, and 100 epochs were iterated. In the training process, the learning rate (LR) was adjusted dynamically, and the learning rate was 0.8 times the current learning rate after 20 epochs.

**Figure 4:**
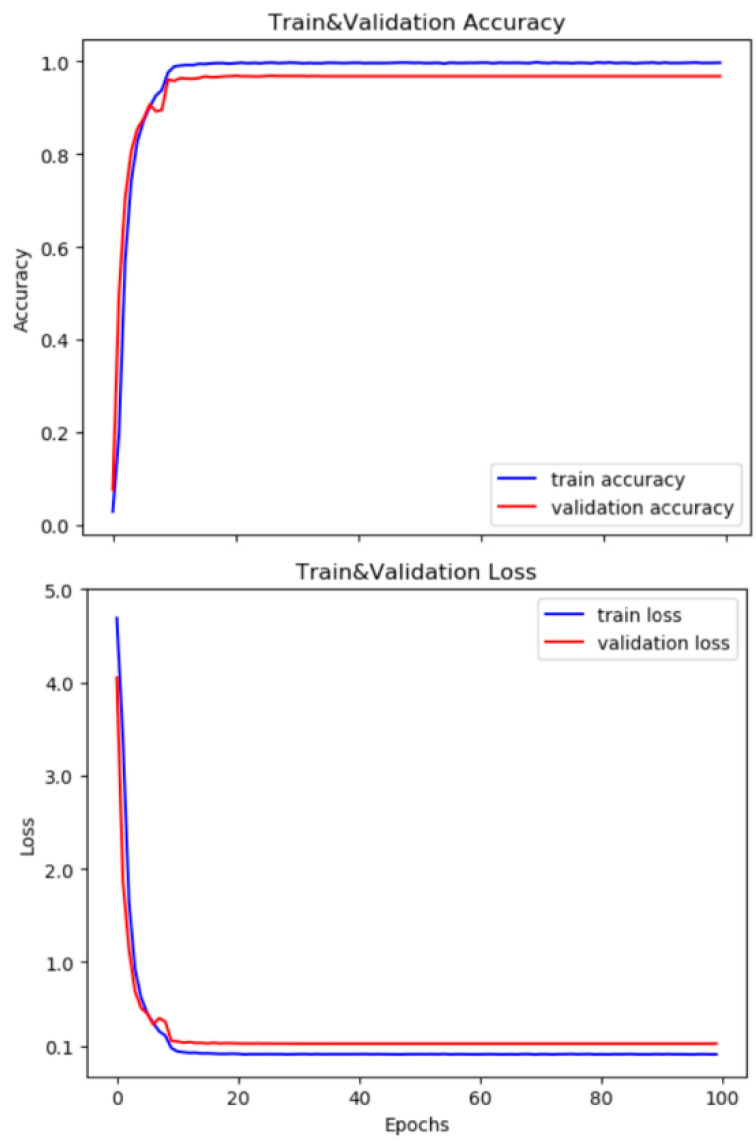
Accuracy and loss curves of CK+ dataset during training.

The accuracy and loss curves of the training and verification sets are shown in Figure 4. As can be seen, the accuracy of the network model gradually improved, and the value of the loss function was reduced gradually.

### 4.4. Visualization of Thermal Diagram

**Figure 5:**
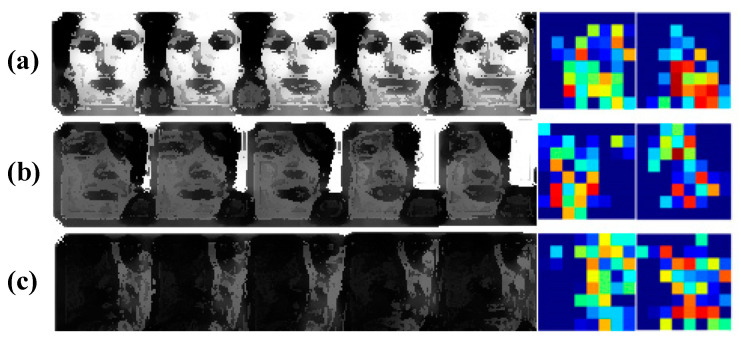
Visualization of hybrid attention module output features (weight increases from blue to red: dark blue–light blue correspond to 0.1–0.4, light blue–yellow correspond to 0.4–0.7, yellow–red correspond to 0.7–1). In the first five columns, the subfigures (**a**–**c**) show image sequences under (i) the ideal condition, (ii) the facial deflection condition, and (iii) the uneven illumination condition. We note that the resolution of the images in these five columns is reduced to protect the privacy. Those images are plotted with the purpose of displaying the profiles of eyes, nose, and mouth, which vary with facial expression changes generally. In addition, the second last column and the last column visualize the attention weights with self-attention and hybrid attention, respectively.

To get a better understanding of the proposed method, we visualize the features output from the hybrid attention module in Figure 5, which shows the distribution of the hybrid attention weight after feature visualization. As can be seen, the weight increases gradually from blue to red. In Figure 5a, the eyes hardly change, and the expression changes are concentrated in the mouth region, which has the highest weight distribution. Figure 5b shows a sequence of facial deflections with facial expressions changing in the mouth and eye regions. For uneven illumination samples (Figure 5c), the hybrid attention module also provides good weight allocation. Figure 5 shows that the hybrid attention module has a good anti-interference effect on FER under uneven illumination and facial deflection conditions. Comparing the last two columns in Figure 5, from the red dots that indicate the concentrated areas, it can be seen that the proposed hybrid attention module (i.e., the last column) performs better than the self-attention module (i.e., the second to last column). To be specific, the attention weights with the hyper-attention module concentrate on the areas that express strong emotions: (1) under the ideal condition, the mouth area is concentrated, which are highlighted by red dots in Figure 5a; (2) under the latter two conditions (i.e., the facial deflection condition and the uneven illumination condition), the eye and mouth areas are concentrated, see red dots in both Figure 5b,c. In contract, the attention weights with self-attention module cannot properly concentrate on the areas that can express strong emotion. Take the uneven illumination condition as an example; there is no red dot in either the eye or mouth area, which indicates that the proposed hybrid attention module is more robust compared with the self-attention module.

**Figure 6:**
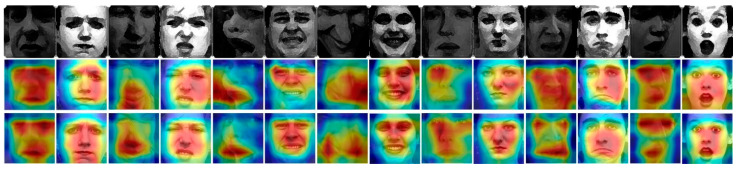
Visual comparison of different facial expressions (using methods with and without attention module). From top to bottom, the expression pictures, the visual results of the method without the attention module, and the visual results of the method with the attention module; From left to right, there are seven expressions (anger, disgust, fear, happy, neutral, sad, and surprised). We note that, the CK+ images (please see the seven even columns in this figure) are from the following seven subjects: s055, s052, s132, s124, s106, s113, and s074, the usage of their images has been approved. Copyright reference: http://www.jeffcohn.net/Resources/ (accessed on 9 March 2021).

We apply the Class Activation Mapping (CAM) [49] to compare the visualization output features of the methods obtained with and without the attention module. As shown in Figure 6, red represents that the current area weight is high, and blue indicates a low area weight. The first row is the input of the expression, i.e., anger, disgust, fear, happy, neutral, sad, and surprised (from left to right). The second row was extracted from the last convolutional layer visualization thermal diagram without the hybrid attention module, and the third row represents the thermal diagram of the method with the hybrid attention module. In each row, every two samples represent the same expression. To be specific, the first one is chosen from AFEW, the second one comes from CK+. After adding the attention module, the method learned the important areas of the facial expression changes, which were primarily concentrated in the mouth, eyes, and nose. Compared to the method without the attention module, the learning area is more accurate, especially on AFEW, and the visualization also shows that the proposed method can learn the attention changes of different expressions.

### 4.5. Experimental Results and Analysis

The average classification accuracy obtained in the experiments on the CK+, AFEW, and Oulu-CASIA datasets is shown in Table 2, Table 3 and Table 4, respectively. 

Table 2 shows the accuracy of our proposed method and state-of-the-art methods [14,18,24,31,33] on the AFEW dataset. On the AFEW dataset, the data come from the natural environment, which is restricted by head deflection, illumination, and blur. Although the challenge of dataset identification is great, the average accuracy of our method reaches 53.44% and has a significant improvement over the other methods [14,18,31,33]. Among other state-of-the-art methods, DenseNet-161 [24] has an accuracy rate of 51.40%, which is 2.04% lower than our method. This also shows that our method is better than other methods for facial expression recognition in natural environments.

Table 3 shows the accuracy of our proposed method and other state-of-the-art methods [17,19,23,27,28,39] on the CK+ dataset where the average accuracy of our method is 98.46%. Compared with the two methods with the highest accuracy, SCAN [39] and FER-IK [23], it is improved by 1.15% and 0.87%, respectively. Table 4 shows the accuracy of proposed method and other state-of-the-art methods [26,27,28,29,39] on the Oulu-CASIA dataset. The average accuracy of proposed method is 87.31%, which is better than DCPN [28] by 1.08%. It is very close to the accuracy of the FaceNet2ExpNet [26] method but 0.39% lower.

We set up two control groups to compare the proposed hybrid attention method to demonstrate the effectiveness of the hybrid attention mechanism and GRU unit. Here, control group 1 was a ResNet50 + GRU cascaded network without the hybrid attention mechanism, and control group 2 was a network with the hybrid attention mechanism embedded in the ResNet50 infrastructure. On the AFEW dataset (Table 2), our method has a significant improvement in accuracy compared to the two control groups, which is 8.39% higher than control group 1 and 2.54% higher than control group 2. On the CK+ dataset (Table 3), our method increased by 4.62% compared to control group 1, and it increased by 1.56% on the basis of control group 2. On the Oulu-CASIA dataset (Table 4), our method improves 7.11% and 2.26% respectively compared with the two control groups. The results prove that the hybrid attention mechanism effectively extracts the dependance between frames, eliminates the interference of irrelevant information in the natural environment, and obtains the attention features of facial expressions. As a result, the recognition rate is improved.

**Figure 7:**
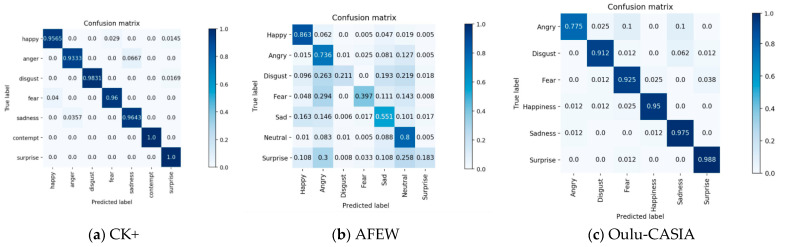
Confusion matrix of networks on CK+, AFEW, and Oulu-CASIA datasets.

Consequently, the confusion matrix and ROC curve of the model were obtained to further evaluate the model. Figure 7 shows the confusion matrix of different datasets trained in the network after five-fold cross-validation. Here, the row of the confusion matrix represents the real label of the current sample, and the column represents the predicted label of the sample. Additionally, the diagonals indicate the correct label. As shown in Figure 7a, the accuracy rate of each label on the CK+ dataset was very high, e.g., the accuracy rate of the contempt and surprise tags was 100%. On the AFEW dataset (Figure 7b), the recognition rate of the happy and neutral expressions was high, which was primarily due to the large number of these expression samples in the AFEW dataset. The recognition rate for disgust and surprise was low because the disgust and surprise expressions are similar, and they are commonly confused with anger. On the Oulu-CASIA dataset (Figure 7c), the recognition rate of the surprise expression was the highest, while that of anger was low. Additionally, significant confusion between anger and sadness was observed.

The receiver operating characteristics (ROC) curve is commonly used for assessing and visualizing the performance of the classification. The False Positive Rate (FPR) on the abscissa of the ROC curve represents the percentage of what are determined as positive cases but not true cases, and the True Positive Rate (TPR) on the ordinate represents the percentage of what are determined as positives cases that are also true cases. The area under the ROC curve (AUC) is a performance indicator that measures the pros and cons of an algorithm. Generally, an AUC area of 0.9 or more is excellent, 0.8 or more is good, and 0.7 or more is medium. In our study, the ROC curve and AUC area were drawn for the three datasets to further evaluate network performance.

**Figure 8:**
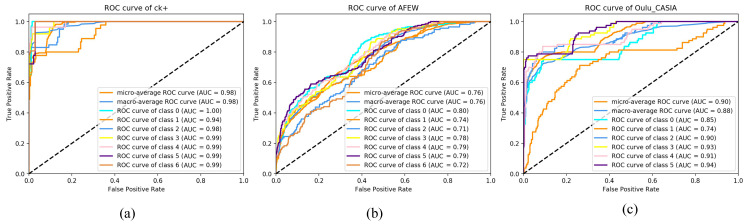
Multiclass receiver operating characteristic (ROC) curves and their area under the ROC curve (AUC) areas on CK+, AFEW, and Oulu-CASIA datasets. In each subfigure, there are either nine or eight lines plotted with different colors, including (i) two lines that represent the macro-average ROC curve and the micro-average ROC curve of the dataset, and (ii) either seven or six lines that represent the corresponding ROC curve for each expression in the dataset. To be specific, in the subfigure (**a**) (i.e., CK+ dataset), classes 0–6 correspond to the expressions of Happy, Anger, Disgust, Fear, Sadness, Contempt, and Surprise, respectively; In subfigure (**b**) (i.e., AFEW dataset), classes 0–6 correspond to the expressions of Happy, Angry, Disgust, Fear, Sad, Neutral, and Surprise respectively; in the subfigure (**c**) (i.e., Oulu-CASIA dataset), classes 0–5 correspond to the expressions of Anger, Disgust, Fear, Happiness, Sadness, and Surprise respectively.

As shown in Figure 8, on the CK+ dataset (Figure 8a), the macro average and micro average of the AUC area of the seven categories all reached 0.98, which proves that the network demonstrated good performance. Similarly, the ROC curve and AUC area on the Oulu-CASIA dataset (Figure 8c) performed well. On the AFEW dataset (Figure 8b), the average area of AUC was 0.76, which also proves the reliability of the model under natural conditions.

## 5. Conclusions

This paper has proposed a cascade network for FER comprising a spatial feature extraction module, a hybrid attention module, and a temporal feature extraction module. In the proposed method, the ResNet50 structure is used to extract spatial features, and the spatial features of facial expressions are input to the hybrid attention module to obtain the fusion features of facial expressions. In addition, based on experimentation, the GRU network structure with 128 neural units in the single-layer hidden layer is employed to extract the temporal feature. In the verification stage of the experiment, the proposed method has been evaluated on three publicly available databases, CK+, Oulu-CASIA, and AFEW. Among them, the first two datasets are experimentally collected, and the last one comes from the natural environment. Then, we achieved recognition accuracies of 98.46%, 87.31%, and 53.44%, respectively, which demonstrate that it is effective to embed the hybrid attention module into convolutional and cyclic neural networks compared to state-of-the-art methods; especially, greater than 2% performance improvement is obtained on the AFEW dataset. The result proves that the hybrid attention mechanism can obtain more effective performance in the natural environment. 

## Figures and Tables

**Table 1:** ResNet50 structure.

Layer Type	Layer Setting	Output Shape	Trainable Parameters
Block_conv1	7 × 7, 64, stride 2	(112, 112, 64)	9508
Block_pool1	3 × 3, max pool stride 2	(56, 56, 64)	0
Block_conv2	$\left[\begin{array}{c} \begin{array}{c} 1\times 1,\;64\\ 3\times 3,\;64\\ 1\times 1,\;256 \end{array} \end{array}\right]\times 3$	(56, 56, 128)	248, 832
Block_conv3	$\left[\begin{array}{c} \begin{array}{c} 1\times 1,\;128\\ 3\times 3,\;128\\ 1\times 1,\;512 \end{array} \end{array}\right]\times 4$	(28, 28, 256)	1, 318, 144
Block_conv4	$\left[\begin{array}{c} \begin{array}{c} 1\times 1,\;128\\ 3\times 3,\;128\\ 1\times 1,\;512 \end{array} \end{array}\right]\times 6$	(14, 14, 512)	7, 492, 096
Block_conv5	$\left[\begin{array}{c} \begin{array}{c} 1\times 1,\;128\\ 3\times 3,\;128\\ 1\times 1,\;512 \end{array} \end{array}\right]\times 3$	(7, 7, 2048)	14, 439, 424
Block_pool2	average pool	(1, 1, 2048)	0
Drop out	0.5	(1, 1, 2048)	0

**Table 2:** Comparison of methods on the AFEW (Acted Facial Expressions in the wild) dataset.

Method	Accuracy
Mode variational LSTM [31]	48.83%
MRAN [14]	49.01%
CNN-BLSTM [33]	49.09%
FAN [18]	51.18%
DenseNet-161 [24]	51.40%
Proposed Method *w*/*o* Attention	45.05%
Proposed Method *w*/*o* GRU	50.90%
Proposed Method	53.44%

**Table 3:** Comparison of methods on CK+ dataset.

Method	Accuracy
LOMo [27]	92.00%
3DIR + landmarks [19]	93.21%
Multiple Attention Network [17]	96.28%
SCAN [39]	97.31%
Inception-w [28]	97.10%
FER-IK [23]	97.59%
Proposed Method *w*/*o* Attention	93.84%
Proposed Method *w*/*o* GRU	96.90%
Proposed Method	98.46%

**Table 4:** Comparison of methods on Oulu-CASIA dataset.

Method	Accuracy
LOMo [27]	74.00%
PPDN [29]	84.59%
SCAN [39]	86.56%
DCPN [28]	86.23%
FaceNet2ExpNet [26]	87.70%
Proposed Method *w*/*o* Attention	80.20%
Proposed Method *w*/*o* GRU	85.05%
Proposed Method	87.31%

## Data Availability

Three open access datasets (AFEW, CK+, Oulu-CASIA) are used in our study. Their links are as follows, https://cs.anu.edu.au/few/AFEW.html (accessed on 9 March 2021), http://www.jeffcohn.net/Resources/ (accessed on 9 March 2021), https://www.oulu.fi/cmvs/node/41316 (accessed on 9 March 2021).
